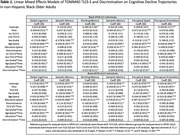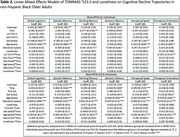# Genetic and Social Determinants of Cognitive Decline in Black Older Adults: Exploring the Role of APOE‐TOMM40‐‘523 Haplotypes, Discrimination, and Loneliness

**DOI:** 10.1002/alz70855_105774

**Published:** 2025-12-23

**Authors:** Katelyn Elizabeth Mooney, Cristian O Mojica, Courtney Thomas Tobin, Lauren L Brown, Tanisha G Hill‐Jarrett, Paris AJ Adkins‐Jackson, Chima Ezeh, Patrick J. Lao, Elizabeth Rose Mayeda, April Thames, Lisa L. Barnes, Kacie D Deters

**Affiliations:** ^1^ University of California, Los Angeles Neuroscience Interdepartmental Program (NSIDP), Los Angeles, CA, USA; ^2^ University of California, Los Angeles, Los Angeles, CA, USA; ^3^ University of California Los Angeles, Community Health Sciences Department, Los Angeles, CA, USA; ^4^ Leonard Davis School of Gerontology, University of Southern California, Los Angeles, CA, USA; ^5^ University of California, San Francisco, San Francisco, CA, USA; ^6^ Columbia University Mailman School of Public Health, New York, NY, USA; ^7^ Columbia University Irving Medical Center, New York, NY, USA; ^8^ Taub Institute for Research on Alzheimer's Disease and the Aging Brain, Vagelos College of Physicians and Surgeons, Columbia University, New York, NY, USA; ^9^ Taub Institute for Research on Alzheimer's Disease and the Aging Brain, New York, NY, USA; ^10^ UCLA Fielding School of Public Health, University of California, Los Angeles, CA, USA; ^11^ Rush Alzheimer's Disease Center, Rush University Medical Center, Chicago, IL, USA; ^12^ University of California Los Angeles, Los Angeles, CA, USA

## Abstract

**Background:**

Social factors such as discrimination and loneliness predict cognitive decline and are commonly reported by Black Americans. *TOMM40*, a genetic marker adjacent to *APOE*, is also linked to cognition. Previous work found Black Americans with *APOE*‐ε3/ε3 and with two copies of the short (S) variant of *TOMM40*‐‘523 have a faster rate of cognitive decline, while the presence of ‘523‐S in Black *APOE*‐ε4+ carriers is related to slower cognitive decline. The extent to which these social and biological factors may work together to influence cognition in Black older adults is unknown. We investigated whether discrimination and loneliness impact the effect of *TOMM40*‐‘523‐S on cognitive decline.

**Method:**

This study included (*N* = 436) dementia‐free non‐Hispanic Black participants from the Minority Aging Research Study, Rush Memory and Aging Project, and Rush African American Clinical Core (Mean_age_=73.1±6.24; Mean_education_=14.7±3.23; Female=80.7%). Linear mixed effect models examined the effects of *TOMM40*‐‘523‐S (0, 1, or 2 copies) within *APOE* genotype (ε3/ε3 or ε4+) and social factors (discrimination, loneliness) on baseline composite measures of global cognition and five cognitive domains. Discrimination was modeled as a dichotomous variable (none vs. presence), while loneliness was continuous. Covariates included age, education (years), and sex/gender, and interactions with time from baseline. Average years of follow‐up was 9.5 years.

**Result:**

Neither discrimination nor loneliness influenced the effect of ‘523‐S on cognitive decline for the global composite or in specific domains. In *APOE*‐ε4+ carriers (*N* = 220), discrimination was associated with a faster rate of decline in working memory (β=‐0.022, S.E.=0.011, *p* = 0.044) and perceptual speed (β=‐0.022, S.E.=0.014, *p* = 0.047) (*Table 1*). Loneliness was not associated with cognitive decline in either *APOE*‐stratified model (ε3/ε3: *N* = 269; ε4+: *N* = 167) (*Table 2*). We also observed baseline effects of discrimination in *APOE*‐ε3/ε3 individuals (*N* = 360, *Table 1*), and of loneliness in *APOE*‐ε4+ individuals (*Table 2*).

**Conclusion:**

Social factors did not influence the effect of *TOMM40‐*’523 on cognitive decline, but the effects of discrimination and loneliness on baseline cognition varied by *APOE*‐genotype, and discrimination predicted cognitive decline in *APOE*‐ε4+ carriers only. These findings suggest that *APOE*‐ε4+ carriers may be vulnerable to the negative effects of discrimination on cognitive decline.